# Mirtrons in Human Cancers

**DOI:** 10.3390/onco5010007

**Published:** 2025-02-08

**Authors:** Yi-Ling Chen, Nicholas Pascuzzi, Alejandro Ruiz, Kuan-Hui Ethan Chen

**Affiliations:** 1 Department of Electronic Engineering, National Kaohsiung University of Science and Technology, Kaohsiung 80778, Taiwan; 2 Department of Biological Sciences; Texas Tech University, Lubbock, TX 79409, USA

**Keywords:** mirtrons, drosha independent, species specific regulation, precision targeting

## Abstract

Mirtrons represent a new subclass of microRNAs (miRNAs) that are processed through non-canonical biogenesis pathways. Unlike canonical miRNAs, which require Drosha-mediated cleavage, mirtrons are generated via the splicing of short intronic sequences, bypassing Drosha entirely. While mirtrons are found across a variety of organisms, their conservation between species is relatively low. This evolutionary divergence has resulted in mirtrons acquiring species-specific regulatory functions. In humans, mirtrons remain an understudied group of regulatory RNAs. However, emerging evidence highlights their critical roles in cancer biology. These small RNAs influence a range of oncogenic processes, including tumor initiation, progression, metastasis, and resistance to therapy. By directly regulating the expression of oncogenes and tumor suppressor genes, mirtrons serve as key molecular mediators within cellular signaling pathways. What sets mirtrons apart from canonical miRNAs is their unique mode of biogenesis and structural attributes, which reveal alternative regulatory mechanisms that could be exploited in cancer biology. Recent advances in understanding their functions suggest that mirtrons hold significant potential as biomarkers for cancer diagnosis and prognosis. Additionally, their role as modulators of cancer pathways positions them as promising therapeutic targets in precision oncology. This review delves into the growing body of research on mirtrons, focusing on their biogenesis, biological roles, and implications in cancer. By emphasizing their distinct features and clinical relevance, it aims to provide a comprehensive perspective on the potential applications of mirtrons in advancing cancer diagnostics and therapeutics.

## Simple Summary

Mirtrons are a unique class of regulatory RNA molecules that merge the splicing and miRNA biogenesis pathways, playing critical roles in post-transcriptional gene regulation. Discovered relatively recently, mirtrons bypass the canonical Drosha-mediated miRNA processing step, instead deriving from spliced introns. They have been implicated in diverse biological processes, including development, immune regulation, and cancer progression. Unlike canonical miRNAs, mirtrons exhibit low evolutionary conservation and tissue-specific regulatory roles, complicating cross-species functional studies. Emerging research highlights their involvement in epigenetic modulation, metabolic reprogramming, and immune evasion, underscoring their biological versatility. In cancer, mirtrons influence key pathways such as epithelial-to-mesenchymal transition, tumor metabolism, and therapy resistance. Despite the identification of 479 human mirtrons, many remain uncharacterized. Advances in RNA profiling and interdisciplinary approaches promise to deepen understanding of mirtrons’ functional diversity and their potential as biomarkers and therapeutic targets, heralding novel insights into RNA biology and precision medicine.

## 1. Introduction

MicroRNAs (miRNAs) are small, non-coding RNAs that play crucial roles in regulating gene expression across diverse biological processes. Since their discovery in the 1990s [1,2], miRNAs have been recognized for their evolutionary conservation and widespread presence across species, from simple organisms to mammals [3,4]. Despite extensive research on canonical miRNAs, a growing body of evidence has revealed alternative classes of small RNAs with distinct biogenesis pathways and unique regulatory functions [5–7]. Among these, mirtrons stand out as a newly identified subclass of miRNAs with non-canonical origins and specialized roles.

What sets mirtrons apart is their distinctive biogenesis pathway and their potential relevance to human diseases. Unlike canonical miRNAs, which rely on the Drosha-DGCR8 complex for cleavage during their maturation process, mirtrons bypass this step entirely [8]. Instead, they originate from short intronic sequences that are spliced out during mRNA processing. Following splicing, the introns undergo debranching and are folded into premiRNA hairpin structures, which are subsequently integrated into the miRNA maturation pathway downstream, beginning with export by Exportin-5 and cleavage by Dicer [9]. This alternative biogenesis pathway not only distinguishes mirtrons from canonical miRNAs but also highlights their potential regulatory flexibility within the broader small RNA landscape. The fact that mirtrons bypass Drosha-mediated cleavage opens up possibilities for their expression and regulation under conditions where canonical miRNA processing may be impaired or altered. Additionally, their direct derivation from spliced introns links them intrinsically to the splicing machinery, further underscoring their unique relationship to gene expression and RNA processing.

The connection between mirtrons and human diseases adds an additional layer of intrigue to their study. Emerging research has demonstrated that mirtrons are differentially expressed in various pathological conditions, including cancers and other stress-related diseases [10–14]. This suggests that mirtrons may have unique roles in cellular stress responses, disease progression, or therapeutic resistance. Their unconventional biogenesis and potential links to disease highlight mirtrons as an exciting area of exploration, offering deeper insights into the complexity and adaptability of small RNA-mediated regulation.

What makes mirtrons even more compelling is their ability to evade degradation in diseased cells, such as transformed cancer cells. While most spliced introns are rapidly degraded in normal cells [15,16], mirtrons or their intronic precursors somehow escape this fate in disease contexts [10]. The mechanism underlying this bypass of intron degradation remains completely unknown, but it raises important questions about how mirtron processing is regulated in pathological conditions. The accumulation of mirtrons in diseased cells, coupled with their distinct biogenesis pathway, opens the door to targeting mirtrons as potential therapeutic agents, further emphasizing their significance in RNA biology and disease research.

Although mirtrons have been identified in a variety of organisms, their conservation between species is relatively low, reflecting evolutionary divergence that has endowed them with species-specific regulatory functions [17]. In humans, mirtrons are an emerging area of study, particularly for their potential roles in health and disease. Recent research suggests that mirtrons may be key modulators of gene expression in cancer biology, where they influence processes such as tumor initiation, progression, metastasis, and therapy resistance [10,11,14,18,19].

This review explores the unique features of mirtrons, including their biogenesis, evolutionary context, functional diversity, and emerging relevance in human cancers. By highlighting the distinct characteristics of mirtrons, this discussion aims to provide insights into their potential applications as biomarkers and therapeutic targets in precision medicine.

## 2. Generation of Canonical miRNAs and Mirtrons

The biogenesis of mirtrons and canonical miRNAs involves distinct pathways, reflecting fundamental differences in their generation processes. These differences highlight the unique regulatory potential of mirtrons compared to canonical miRNAs. Below is a comparison of their respective biogenesis mechanisms.

**Figure 1.**
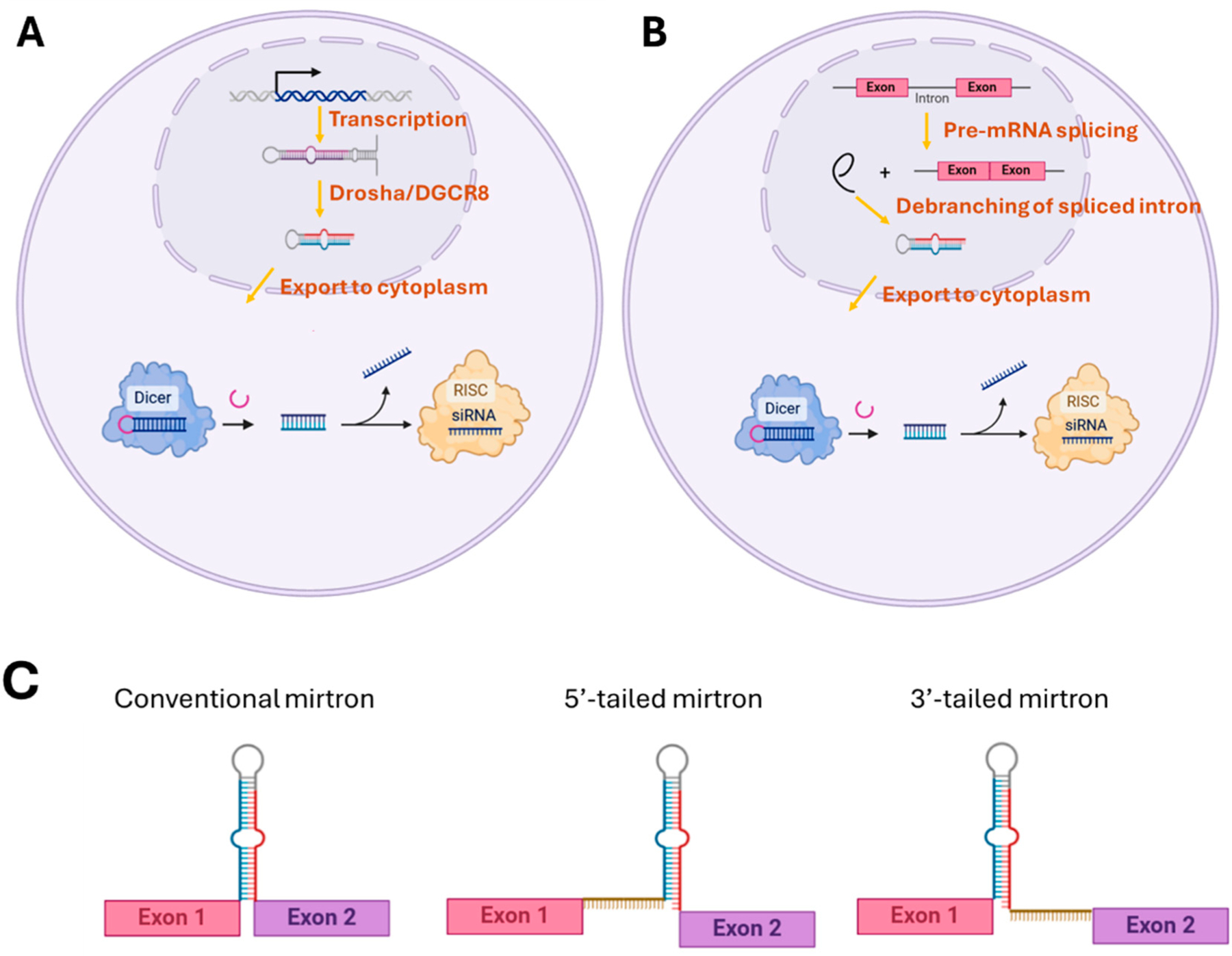
A diagram illustrating the biogenesis pathways of canonical miRNAs (**A**) and mirtrons (**B**), along with the subclassification of mirtrons into conventional, 5′-tailed, and 3′-tailed types (**C**).

Canonical miRNAs are generated through a well-characterized, multi-step process (Figure 1A). Typically, canonical miRNAs are transcribed by RNA polymerase II as primary miRNAs (pri-miRNAs), which are long, hairpin-structured precursors. The Microprocessor complex, consisting of Drosha (a ribonuclease III enzyme) and its cofactor DGCR8, processes the pri-miRNA in the nucleus. This step excises a smaller, ~70-nucleotide precursor miRNA (pre-miRNA) with a characteristic stem-loop structure [20]. The pre-miRNA is transported to the cytoplasm by Exportin-5 in a Ran-GTP-dependent manner [21]. In the cytoplasm, the RNase III enzyme Dicer processes the pre-miRNA into a mature miRNA duplex (~22 nucleotides) [22]. One strand of the duplex, known as the guide strand, is incorporated into the RNA-induced silencing complex (RISC), where it guides the silencing of target mRNAs [22].

Mirtrons follow a non-canonical biogenesis pathway that bypasses the Drosha cleavage step, relying instead on splicing and debranching processes (Figure 1B). Mirtrons are transcribed as part of the host gene’s intronic sequences, typically through RNA polymerase II. The intronic sequence containing the mirtron is spliced out during the pre-mRNA splicing process [9]. During splicing, mirtrons can be classified into three subclasses based on how splicing defines their pre-miRNA hairpin ends (Figure 1C). In conventional mirtrons, both hairpin ends are generated through splicing. In contrast, tailed mirtrons have only one hairpin end defined by splicing. For instance, in 3′-tailed mirtrons, the 5′ hairpin end is created by splicing at the 5′ splice site, while an unstructured region extending from the 3′ splice site forms a 3′ tail. This tail structure can be trimmed by the RNA exosome [23]. Similarly, 5′-tailed mirtrons have their 3′ hairpin ends defined by the 3′ splice site, with an unstructured region extending from the 5′ splice site that generates a 5′ tail. While the mechanism for trimming the 5′ tail remains unclear, a recent study by Farid Zia et al. suggests that RNaseP may play a role in this process [24]. The excised intronic lariat structure is debranched by a debranching enzyme, resulting in a linear intronic RNA with a hairpin-like structure [9,25,26]. Similar to pre-miRNAs, mirtrons are exported to the cytoplasm by Exportin-5 [27]. Once in the cytoplasm, Dicer processes the hairpin-structured mirtron into a mature miRNA duplex. The guide strand of the mirtron-derived miRNA duplex is loaded into RISC to mediate gene silencing [22,28].

One of the key distinctions between mirtrons and canonical miRNAs lies in the involvement of splicing during their biogenesis. While pre-mRNAs typically contain multiple introns, not all introns have the potential to generate mirtrons. According to studies by Wen et al., there are 478 and 488 confirmed mirtrons in humans and mice, respectively [17]. Additionally, mirtronDB, which compiles through an extensive literature review, lists 480 confirmed human mirtrons with an additional 105 potential candidates and 482 confirmed mouse mirtrons with 35 potential candidates [29]. Among the mirtron precursors confirmed through Wen’s work or mirtronDB, current evidence suggests that mirtron biogenesis predominantly occurs from short introns [29–31]. Although the precise size range of introns capable of producing mirtrons is not conclusively defined in the literature, our analysis of 488 confirmed mouse mirtrons, as compiled from both Wen’s study and mirtronDB, offers valuable insights. Among these, 176 mirtrons are derived from introns shorter than 150 base pairs (bp), and another 93 originate from introns measuring between 150 and 500 bp. Notably, a significant portion, 219 mirtrons, are generated from introns larger than 500 bp. The most extreme example is the mirtron mmu-mir-5129, which is derived from an exceptionally large intron of 86,911 bp within the Zeb2 gene.

A similar pattern emerges in our other analysis of 480 human mirtrons retrieved from the studies by Wen et al. and mirtronDB [17,29]. Of these, 165 are derived from introns shorter than 150 bp, 110 from introns between 150 and 500 bp, and the remaining mirtrons from introns larger than 500 bp. While not as extreme as mmu-mir-5129, the human mirtron hsa-mir-1527 is generated from a notably large intron of 26,691 bp within the GATAD2A gene. Table 1 summarizes the distribution of mirtrons across introns of varying sizes.

This diversity in intron size highlights the adaptability of mirtron biogenesis and suggests that while shorter introns are generally favored, longer introns may also contribute to mirtron production under specific conditions. Further research is needed to clarify the mechanisms that enable the selection of certain introns for mirtron generation.

In summary, although canonical miRNAs and mirtrons ultimately share common downstream pathways, including Dicer processing and RISC loading, their distinct upstream biogenesis pathways highlight alternative strategies for producing small regulatory RNAs. These variations not only expand the regulatory versatility of miRNAs but also emphasize the evolutionary flexibility of RNA-based gene regulatory mechanisms.

## 3. Evolutionary Conservation of Mirtrons

Mirtrons represent a unique subclass of small RNAs that deviate from the canonical miRNA biogenesis pathway. Unlike canonical miRNAs, which are typically highly conserved across species, mirtrons exhibit variable levels of evolutionary conservation. This variability reflects both their distinct biogenesis mechanisms and their species-specific regulatory roles. Below is an in-depth review of the evolutionary conservation of mirtrons.

Canonical miRNAs often exhibit remarkable conservation across a wide range of taxa, including plants, worms, flies, and mammals. This high level of evolutionary conservation underscores their essential roles in fundamental biological processes, such as development, differentiation, and homeostasis, that have remained largely unchanged over evolutionary time. One of the most notable examples is the let-7 family, which is highly conserved in animals from *Caenorhabditis elegans* to humans [32–34]. The let-7 miRNAs play critical roles in regulating developmental timing [32], cell cycle progression [35], and differentiation by targeting multiple conserved genes involved in these pathways [36,37]. The conservation of let-7 highlights the pivotal roles canonical miRNAs play in maintaining key cellular functions across species. Beyond let-7, other conserved canonical miRNAs further illustrate their evolutionary importance. For instance, miR-1 is found in nematodes, flies, and humans [38], where it plays a pivotal role in muscle development and function. Similarly, the miR-143/145 cluster is conserved among vertebrates [39,40], where it is involved in vascular development, smooth muscle cell regulation, pigmentation in melanocytes and iridophores, craniofacial development, and chondrogenesis. These examples demonstrate the shared regulatory mechanisms that canonical miRNAs contribute to across diverse species, emphasizing their foundational roles in gene expression and organismal biology.

In contrast, mirtrons typically exhibit low conservation across species. A small subset of mirtrons shows conservation among phylogenetically related organisms, particularly within Drosophila and other insects. These conserved mirtrons may perform fundamental regulatory functions similar to those of canonical miRNAs. For example, mirtron-1017 is conserved across insects and plays a role in regulating insect longevity [41]. However, the majority of mirtrons are species-specific or have only limited orthologs in closely related species. To further investigate this, we analyzed the sequences of confirmed mirtrons in mouse (488 identified mirtrons) and human (480 identified mirtrons) retrieved from mirtronDB and the studies by Wen et al. [17,29], as the mouse model is extensively used for preclinical studies. We assessed the conservation between mouse and human mirtrons based on the conservation of the seed sequence and positional alignment after aligning the mirtrons from both species using the UCSC human/hg19 46-way multiple alignment. Consistent with previous reports [17], we identified only 13 putative mirtron “orthologs” shared between humans and mice. Tables 2 and 3 provide details on these orthologs. Although these orthologous mirtrons share some degree of sequence similarity, they are not completely identical, suggesting that their regulatory roles may differ between humans and mice. This limited conservation underscores the evolutionary divergence of mirtrons and supports the idea that they may have evolved to fulfill species-specific or niche-specific regulatory functions.

Several factors account for the limited evolutionary conservation of mirtrons. Unlike canonical miRNAs, mirtron biogenesis depends on the splicing of introns from host genes. Splicing mechanisms and intronic sequences are highly variable across species, significantly impacting the emergence and retention of mirtrons [42]. Even closely related species, such as humans and chimpanzees, show considerable differences in splicing patterns [43]. Furthermore, introns, which serve as precursors for mirtrons, tend to evolve more rapidly than coding regions or conserved non-coding sequences. This inherent susceptibility to evolutionary changes reduces the likelihood of mirtrons being preserved across distant evolutionary timelines. These factors collectively contribute to the species-specific and dynamic nature of mirtron evolution.

In summary, mirtrons exhibit markedly lower evolutionary conservation compared to canonical miRNAs, a difference that underscores their distinct origins and functions. This limited conservation presents challenges for functional annotation and cross-species analysis, necessitating species-specific investigations to elucidate their roles. However, the independent emergence of mirtrons in diverse species suggests their potential to drive the evolution of novel regulatory networks. This adaptability enhances the flexibility of gene expression control, enabling more precise regulation tailored to specific species. Such precision may hold promise for advancing our understanding of gene expression and developing species-specific strategies for disease management.

## 4. Gene Ontology Analyses and Potential Implications of Mirtrons in Human Cancers

**Figure 2.**
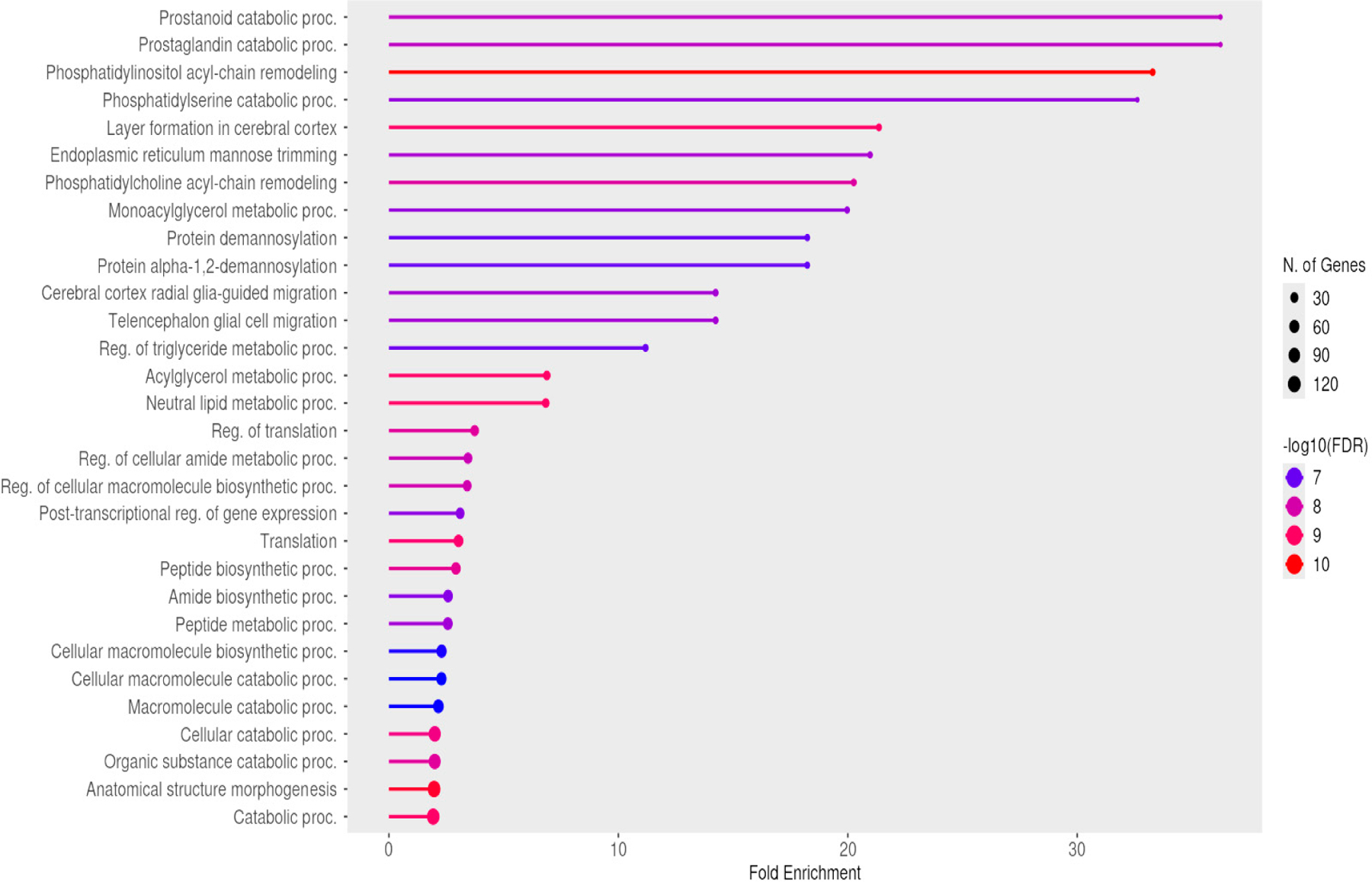
Gene ontology (GO) analysis of loci housing human mirtrons.

As previously discussed, mirtron generation is intrinsically linked to the expression and splicing of their host genes. Consequently, mirtrons are often regarded as byproducts of host gene activity. To better understand the conditions under which human cells generate mirtrons, we conducted a gene ontology (GO) analysis of the genes harboring the 480 confirmed human mirtrons. Figure 2 illustrates the GO analysis results, highlighting the top 30 associated biological processes. A significant proportion of these processes are related to cellular metabolism, cell migration, and morphological changes. To further expand the GO analysis, we included genes harboring currently known cancer-related mirtrons reported in the literature, such as VWA5B2 (hsa-mir-1224), ENSG00000008710 (hsa-mir1225), DHX30 (hsa-mir-1226), LRP1 (hsa-mir-1228), MGAT4B (hsa-mir-1229), GOLGA8A (hsa-mir-1233), NOP56 (hsa-mir-1292), RPS6KA1 (hsa-mir-1976), PHC2 (hsa-mir-3605), SHMT1 (hsa-mir-6778), PISD (hsa-mir-7109), ABCF1 (hsa-mir-877), and SCRIB (hsa-mir-937). As shown in Table 4, these genes are primarily involved in processes such as cell growth, motility, adhesion, and immune regulation, further emphasizing their relevance in cancer development. These findings suggest a potential connection between mirtrons and cancer-related pathways.

Supporting this hypothesis, several studies have documented altered expression of mirtrons specifically in cancers [10,12,44–46]. Mirtrons appear to play roles in cancer cell metabolism, which may contribute to enhanced cellular stemness [12]. In this section, we will review recent advances how mirtrons contribute to tumor progression.

### Regulation of Oncogenes and Tumor Suppressors

Like canonical miRNAs, mirtrons regulate gene expression by binding to complementary sequences in the 3′ untranslated regions (UTRs) of target mRNAs, reducing their stability or inhibiting translation. For example, mirtron hsa-mir-1228 directly targets the tumor suppressor protein TP53, facilitating hepatoma cell proliferation [47,48]. In high-grade serous ovarian cancers, mirtron hsa-mir-937–5p plays a critical role in cancer proliferation by targeting FBXO16, a putative tumor suppressor involved in inhibiting cell proliferation, clonal survival, and invasion [49]. Similarly, in non-small cell lung cancer (NSCLC), the expression of mirtron hsa-mir-937–3p is regulated by c-Myc and promotes processes such as angiogenesis and tumor invasion [50]. Additionally, in breast cancers, the tumor suppressor zinc finger and BTB domain containing 1 (ZBTB1) has been identified as a direct target of mirtron hsa-mir-1229–3p [51].

In contrast to these oncogenic roles, some mirtrons act as tumor suppressors. For instance, mirtron hsa-mir-1292 targets the proto-oncogenic protein DEK, thereby inhibiting gastric cancer proliferation [52]. Furthermore, mirtron expression patterns are not uniformly upregulated across all cancer types. For example, mirtron hsa-mir-1229–3p is overexpressed in breast, pancreatic, and stomach cancers [10,51], while mirtron hsa-mir-1226–3p is upregulated in stomach tumors but downregulated in colorectal tumors [10].

These examples highlight the diverse and context-dependent roles of mirtrons in modulating oncogenic pathways and tumor progression, emphasizing their functional versatility in interacting with specific mRNA targets. Moreover, mirtrons may display tissue-specific splicing patterns, contributing to the regulation of various cancer types.

### Influence on Cancer Cell Metabolism

Cancer cells undergo metabolic reprogramming to support their rapid proliferation and survival. One hallmark of this reprogramming is the Warburg effect, where tumor cells preferentially rely on aerobic glycolysis for energy production, even in the presence of sufficient oxygen, instead of oxidative phosphorylation. ZBTB1 has been shown to suppress glucose uptake and counteract aerobic glycolysis in breast cancer cells [53]. However, ZBTB1 is directly targeted by mirtron hsa-mir-1229–3p, which is upregulated in breast cancers [51]. This interaction likely contributes to the metabolic alterations observed in cancer cells.

Another key feature of cancer metabolism is one-carbon metabolism, which includes the folate and methionine cycles. These pathways generate one-carbon units essential for nucleotide synthesis, methylation, and reductive metabolism, all of which support the high proliferation rate of cancer cells. SHMT1, which encodes cytoplasmic serine hydroxymethyltransferase, plays a critical role in one-carbon metabolism and is naturally suppressed by YWHAE (tyrosine 3-monooxygenase/tryptophan 5-monooxygenase activation protein epsilon) [54]. Interestingly, mirtron hsa-mir-6778–5p targets YWHAE, thereby increasing SHMT1 expression to sustain cancer cell survival and maintain stemness [14].

Lipogenesis in tumor-associated adipocytes is another aspect of metabolic reprogramming. This process involves adipocytes near tumors actively producing new lipids (fatty acids) through lipogenesis, providing readily available energy to tumor cells and fueling their growth and proliferation [55]. In a dietary mouse model, mirtron hsa-mir-1224–5p has been shown to enhance lipogenesis in the liver by suppressing adenosine monophosphate-activated protein kinase (AMPK)-α1, a key regulator of cellular energy homeostasis [56].

Although this study was not conducted in tumor-bearing mice, it suggests that mirtron hsa-mir-1224–5p could similarly affect tumor-associated adipocytes, driving lipogenesis to support tumor metabolism. AMPK, widely recognized as a tumor suppressor, regulates numerous metabolic pathways in cells [57]. Future investigations into the role of mirtron hsa-mir-1224–5p in targeting AMPK may uncover additional insights into its broader metabolic impacts in cancer progression.

### Promotion of Cell Migration and Metastasis

Mirtrons significantly influence cancer cell migration and invasion by regulating genes associated with epithelial-to-mesenchymal transition (EMT), cytoskeletal remodeling, and extracellular matrix interactions. These mechanisms are crucial for metastasis, enabling cancer cells to disseminate to distant tissues. For instance, in colorectal cancer, the expression of mirtron hsa-mir-1226–5p has been linked to enhanced tumor cell migration and a heightened EMT process [58]. In triple-negative breast cancer (TNBC), TIMP3 functions as a tumor suppressor by strongly inhibiting angiogenesis, metalloprotease activity, and cellular migration. However, the overexpression of mirtron hsa-mir-877–5p in TNBC cells downregulates TIMP3, thereby promoting tumor metastasis [59]. Interestingly, mirtron hsa-mir-877–5p demonstrates contrasting roles in other cancers, where it suppresses tumor invasion in prostate and bladder cancers, highlighting its context-specific regulatory functions [60,61].

Moreover, several human mirtrons are secreted via exosomes, enabling them to travel to distant tissues and exert systemic effects. Exosomal mirtrons have been implicated in regulating tumor migration, invasion, and metastasis. For example, mirtron hsa-mir-1228–5p has been identified in exosomes derived from small cell lung cancer cells, where it promotes tumor cell migration by targeting DUSP22 [62]. These examples highlight the intricate and multifaceted roles of mirtrons in cancer progression, emphasizing their potential as both therapeutic targets and diagnostic biomarkers.

### Resistance to Therapy

Mirtrons may contribute to therapy resistance by enabling cancer cells to evade the cytotoxic effects of chemotherapy or targeted treatments, ultimately leading to treatment failure and disease recurrence.

In estrogen/estrogen receptor-sensitive breast cancers, therapies such as selective estrogen receptor modulators (e.g., tamoxifen) and estrogen receptor antagonists (e.g., fulvestrant) are commonly employed. However, resistance to these therapies has been reported. Differential RNA profiling analyses of human breast cancer cells have identified correlations between specific miRNAs, including mirtrons, and hormone therapy resistance [63]. Notably, mirtron hsa-mir-1226 has been linked to increased resistance to tamoxifen, while mirtron hsa-mir-1228 is associated with resistance to both fulvestrant and tamoxifen [63]. It is widely accepted that tamoxifen resistance is partially driven by abnormal estrogen receptor expression, dysregulation of the PI3k/Akt/mTOR pathway, or the bypassing of the Cyclin/CDK4/6 pathway. In a separate study, Kim et al. showed that the mirtron hsa-mir-1233–3p contributes to tamoxifen resistance by modulating the PI3k/Akt/mTOR pathway, specifically targeting PIK3R1, a subunit of PI3k [64]. Similarly, research by Torrisi et al. identified a panel of seven small RNAs, including hsa-mir-1233, that were significantly associated with resistance to Palbociclib (a CDK4/6 inhibitor) and endocrine therapy in metastatic breast cancer patients [65]. The development of therapeutic resistance is likely due to the direct targeting of the PI3K/Akt/mTOR pathway, cell cycle regulators, and autophagy by these seven small RNAs [65]. In contrast, the status of hsamiR-1233–3p as a bona fide mirtron has been questioned [66]. Schamberger et al. showed that hsa-miR-1233–3p did not suppress gene expression but instead increased it, using an artificial reporter construct followed by an antisense sequence to the mirtron. While there are known examples of small RNAs positively regulating gene translation [67–70], the precise mechanism of action for miR-1233 requires further investigation. Exosomal mirtrons play a role in mediating drug resistance. For instance, exosomes derived from tumor-associated fibroblasts contain mirtron hsa-mir-1228–3p, which enhances liver cancer cell resistance to sorafenib by directly targeting placenta-associated 8 (PLAC8) [71]. PLAC8 directly interacts with Akt, inhibiting its phosphorylation and activation, thereby acting as a negative regulator of the PI3k/Akt pathway. The mirtron hsa-mir-1228–3p targets PLAC8, thereby activating the PI3K/Akt pathway and contributing to the development of resistance. Similarly, higher expression levels of mirtron hsa-mir-1976 in liver and pancreatic cancers have been associated with increased chemoresistance, although the underlying mechanism remains unclear [72].

These findings highlight the significant role of mirtrons in driving therapy resistance, making them potential targets for overcoming treatment challenges in cancer.

### Immune Evasion

Emerging evidence indicates that mirtrons can influence the tumor microenvironment by modulating immune checkpoint molecules or cytokines. By reshaping the immune landscape, mirtrons may help cancer cells evade immune detection.

In colorectal cancer, hsa-mirtron-1226–5p promotes immune evasion by targeting IRF1, which increases M2 macrophage polarization, leading to elevated TGF-β production and an immunosuppressive microenvironment [58]. Similarly, in ovarian cancer, overexpression of the mirtron hsa-mir-1225–5p facilitates M2 macrophage accumulation by directly targeting toll-like receptor 2 (TLR2) [73]. However, mirtron hsa-mir-1225–5p has also been reported to exhibit tumor-suppressive roles in certain cancers, highlighting its context-dependent functions [74,75].

Not all mirtrons contribute to immune evasion. For instance, mirtron hsa-mir-7109 has been shown to target the immune checkpoint molecule siglec-15, restoring immune surveillance and promoting anti-tumor immunity [76].

These findings underscore the diverse roles of mirtrons in modulating the immune microenvironment, presenting them as potential therapeutic targets to manipulate immune responses in cancer.

### Epigenetic Regulation

Mirtrons have also been implicated in epigenetic regulation, with evidence suggesting their expression can be modulated by DNA methylation and RNA modifications, highlighting their potential role in cancer development.

A study investigating epigenetic silencing in urothelial carcinoma (UCC) revealed that mirtrons are often located near CpG islands, which makes them prone to hypermethylation in cancer. This hypermethylation may lead to their epigenetic silencing and loss of function. For instance, miR-1224, located within the VWA5B2 gene and near the p63 tumor suppressor gene, was found to be hypermethylated in UCC. The proximity of miR-1224 to these key regulatory genes suggests a potential link between its silencing and tumorigenesis, hinting at another layer of mirtron involvement in cancer biology [12]. In addition to potential epigenetic silencing, CpG island methylation has been shown to influence RNA splicing. Methylation of CpG sites within or near exons can recruit methyl-CpG binding protein 2 (MeCP2), which interact with splicing factors to regulate exon inclusion or skipping during RNA splicing [77]. Given that mirtron biogenesis relies heavily on splicing and the expression of host genes, the epigenetic regulation of host gene silencing and exon skipping could, therefore, affect mirtron generation.

Additionally, post-transcriptional modifications, such as N6-methyladenosine (m6A), may influence mirtron function. A study by Wang demonstrated that m6A modification can impact the activity of mirtron hsa-mir-3605–5p in esophageal squamous cell carcinoma, contributing to tumorigenesis [78]. These modifications may alter mirtron stability, processing, or target interactions, further extending their regulatory influence in the epigenetic landscape of cancer.

Together, these findings highlight the multifaceted role of mirtrons in epigenetic regulation and underscore their potential as biomarkers for cancer diagnosis and as targets for epigenetic therapy.

## 5. Conclusions

Mirtron research has revealed a fascinating class of regulatory RNA molecules that integrate the splicing and miRNA pathways, offering valuable insights into the complexities of post-transcriptional gene regulation. Since their discovery, mirtrons have been implicated in a wide range of biological processes, including development, immune response modulation, and cancer progression. Their unique biogenesis, species-specific roles, and emerging involvement in epigenetic and metabolic regulation underscore their versatility and highlight their potential as novel biomarkers and therapeutic targets.

As previously discussed, mirtron biogenesis results from gene splicing. In normal cells, most spliced introns are quickly degraded [15,16]. However, it is intriguing that mirtrons, derived from spliced introns, evade degradation and instead proceed to form functional mirtrons. Studies have highlighted that mirtrons are specifically upregulated in cancers, pointing to the possibility of an unknown mechanism that prevents their degradation in cancer cells. Therefore, mirtrons in cancer may represent potential and targeted therapeutic avenues for cancer treatment. Since mirtron biogenesis does not rely on Drosha, targeting mirtrons would likely focus on the tail-trimming process during their maturation. While conventional mirtrons are directly generated by splicing, most confirmed mirtrons are 5′-tailed, with a smaller proportion being 3′-tailed. RNA exosomes and other nucleases play essential roles in trimming these 5′ and 3′ tails. Consequently, these nucleases could serve as potential therapeutic targets to regulate mirtron biogenesis in cancer cells. Additionally, regulation of splicing through mechanisms like epigenetic modifications could provide an alternative approach to controlling mirtron production in cancer.

Although 480 mirtrons have been identified in humans, the field remains nascent, with many mirtrons yet to be studied. A major challenge in mirtron research is the low evolutionary conservation between species, which limits the ability to extrapolate findings across organisms. Moreover, some mirtrons have demonstrated tissue- and cancer-specific regulatory functions, suggesting the existence of context-dependent mechanisms that further complicate their characterization.

As technologies for RNA profiling, epigenetic analysis, and functional assays continue to advance, they will provide researchers with the tools needed to uncover the full spectrum of mirtron functions. Interdisciplinary approaches integrating bioinformatics, molecular biology, and clinical research are likely to accelerate the understanding of mirtrons. These efforts promise to unlock innovative applications in diagnostics, personalized medicine, and targeted therapies, solidifying mirtrons as key players in the landscape of RNA biology and disease management.

## Figures and Tables

**Table 1.** The number (#) of mirtrons derived from introns of different sizes.

Size of Intron	# of Mouse Mirtrons (489)	# of Human Mirtrons (479)
<100 bp	125	115
101–150 bp	52	49
151–250 bp	33	53
251–500 bp	60	57
501–1000 bp	70	68
1001–2500 bp	92	71
2501–5000 bp	27	47
5001–10,000 bp	19	13
10,001–50,000 bp	9	6
>50,000 bp	2	0

**Table 2.** Putative human mirtron orthologs to mouse.

Locus	Sequence	Gene Symbol
hsa-mir-1224	GTGAGGACTCGGGAGGTGGAGGGT	VWA5B2
hsa-mir-1229	GTGGGTAGGGTTTGGGGGAGAGCG	MGAT4B
hsa-mir-3064	TCTGGCTGTTGTGGTGTGCAA	DDX5
hsa-mir-6745–3p	TGGGTGGAAGAAGGTCTGGTT	PACSIN3
hsa-mir-6751	TTGGGGGTGAGGTTGGTGTCTGG	SYVN1
hsa-mir-6767	TCGCAGACAGGGACACATGGAGA	CCNF
hsa-mir-6777	ACGGGGAGTCAGGCAGTGGTGGA	SREBF1
hsa-mir-877	GTAGAGGAGATGGCGCAGGGGACA	ABCF1
hsa-mirtron-1610–5p	CCAGGGTGGGATGAGGCTTGGGA	FCHO1
hsa-mirtron-1259–3p	TAAGCTCCCTGCCTCCTGTAG	CAD
hsa-mirtron-1488–3p	TGACCACCGTGCCTCTCCCAG	TRIOBP
hsa-mirtron-1568–5p	GTGTGGAGGGAATGGGGGCTATGT	PKN3
hsa-mirtron-1559–3p	CTGAGCCCTGTCCTCCCGCAG	GOLGA2

**Table 3.** Putative mouse mirtron orthologs to human.

Locus	Sequence	Gene Symbol
mmu-mir-1224	GTGAGGACTGGGGAGGTGGA	Vwa5b2
uc007iry.12	TGTGTGGGCTGGGCTTTTGG	Mgat4b
mmu-mir-3064	TCTGGCTGTTGTGGTGTGCAA	Ddx5
uc008kvn.5	TGCGGGCCTGAGTGGAAGGCAGT	Pacsin3
mmu-mir-6988	TGGGGTGGAGAGCTGAGGCCCAG	Syvn1
mmu-mir-5134	TTGGCAGAAAGGGCAGCTGTGA	Ccnf
mmu-mir-6921	TGAGGGGCATGAGGTAGGAAGC	Srebf1
mmu-mir-877	GTAGAGGAGATGGCGCAGGGGACA	Abcf1
uc009met.10	TGGGAACAGGAACAGCCTGTGG	Fcho1
uc008wwz.19	ACTGACCCTCCTGTCCCTGCAG	Cad
mmu-mir-6956	TGACCGGCCTATCCTCTCAG	Triobp
uc008jba.8	GTGAGGAGAGGGCTGGGCTGA	Pkn3
uc008jev.23	CACCTGCCTGCCGTCTCCACAG	Golga2

**Table 4.** Genes housing cancer related mirtrons are grouped by functional categories defined by high-level GO terms.

N	High Level GO Category	Genes
11	GO:0006950 response to stress	SCRIB LRP1 RPS6KA1 ABCF1
11	GO:0009893 positive regulation of metabolic process	RPS6KA1 ABCF1 SCRIB LRP1
4	GO:0009056 catabolic process	LRP1 SHMT1 PISD
4	GO:0009653 anatomical structure morphogenesis	SCRIB RPS6KA1 LRP1
4	GO:0042221 response to chemical	SHMT1 SCRIB LRP1
4	GO:0044085 cellular component biogenesis	SHMT1 NOP56 DHX30
4	GO:0065009 regulation of molecular function	LRP1 RPS6KA1 SCRIB
3	GO:0008283 cell population proliferation	SCRIB RPS6KA1
3	GO:0009719 response to endogenous stimulus	SHMT1 LRP1
2	GO:0002376 immune system process	SCRIB LRP1
2	GO:0040007 growth	RPS6KA1
2	GO:0040011 locomotion	SCRIB LRP1
2	GO:0016049 cell growth	RPS6KA1
2	GO:0023051 regulation of signaling	SCRIB LRP1
2	GO:0032879 regulation of localization	SCRIB LRP1
2	GO:0033036 macromolecule localization	SCRIB LRP1
2	GO:0040008 regulation of growth	RPS6KA1
2	GO:0048870 cell motility	SCRIB LRP1
2	GO:0050793 regulation of developmental process	RPS6KA1
2	GO:0051094 positive regulation of developmental process	RPS6KA1
2	GO:0051234 establishment of localization	LRP1 SCRIB
2	GO:0051239 regulation of multicellular organismal process	SCRIB LRP1
2	GO:0051240 positive regulation of multicellular organismal process	SCRIB LRP1
2	GO:0051641 cellular localization	SCRIB LRP1
2	GO:0051674 localization of cell	SCRIB LRP1
1	GO:0000003 reproduction	PHC2
1	GO:0022414 reproductive process	PHC2
1	GO:0002252 immune effector process	LRP1
1	GO:0002682 regulation of immune system process	SCRIB
1	GO:0002683 negative regulation of immune system process	SCRIB
1	GO:0003006 developmental process involved in reproduction	PHC2
1	GO:0003008 system process	LRP1
1	GO:0006955 immune response	LRP1
1	GO:0007155 cell adhesion	SCRIB
1	GO:0009605 response to external stimulus	SCRIB
1	GO:0016080 synaptic vesicle targeting	SCRIB
1	GO:0019953 sexual reproduction	PHC2
1	GO:0023057 negative regulation of signaling	LRP1
1	GO:0030155 regulation of cell adhesion	SCRIB
1	GO:0032504 multicellular organism reproduction	PHC2
1	GO:0040012 regulation of locomotion	LRP1
1	GO:0040013 negative regulation of locomotion	LRP1
1	GO:0042330 taxis	SCRIB
1	GO:0045321 leukocyte activation	SCRIB
1	GO:0048583 regulation of response to stimulus	LRP1
1	GO:0048609 multicellular organismal reproductive process	PHC2
1	GO:0048646 anatomical structure formation involved in morphogenesis	SCRIB
1	GO:0065008 regulation of biological quality	SCRIB
